# The Controversial Definition of Normal Toe Alignment

**DOI:** 10.3390/jcm12103509

**Published:** 2023-05-17

**Authors:** Philipp Schippers, Philipp Drees, Erol Gercek, Felix Wunderlich, Daniel Müller, Christian Ruckes, Alexander Meyer, Stefan Klein, Sebastian Fischer

**Affiliations:** 1 Department of Orthopedics and Traumatology, University Medical Center of the Johannes Gutenberg, University Mainz, 55131 Mainz, Germany; 2 Department of Trauma, Hand, and Reconstructive Surgery, Goethe-Universität Frankfurt am Main, 60596 Frankfurt, Germany; 3 Interdisciplinary Centre for Clinical Trials Mainz, University Medical Center, Johannes Gutenberg University Mainz, 55131 Mainz, Germany; 4 Center for Orthopedics, Spine and Trauma Surgery, St. Josefs-Hospital, 65189 Wiesbaden, Germany; 5 Department of Diagnostic and Interventional Radiology, Berufsgenossenschaftliche Unfallklinik Frankfurt am Main, 60389 Frankfurt, Germany; 6 Department of Foot and Ankle Surgery, Berufsgenossenschaftliche Unfallklinik Frankfurt am Main, 60389 Frankfurt, Germany

**Keywords:** toe alignment, forefoot, parabola, metatarsophalangeal angle, transverse plane

## Abstract

“Normal” and “abnormal” are frequently used in surgical planning and to evaluate surgical results of the forefoot. However, there is no objectifiable value of metatarsophalangeal angles (MTPAs) 2–5 in the dorsoplantar (DP) view with which to objectively evaluate lesser toe alignment. We aimed to determine which angles are considered to be “normal” by orthopedic surgeons and radiologists. Thirty anonymized radiographs of feet were submitted twice in randomized order to determine the respective MTPAs 2–5. After six weeks, the anonymized radiographs and photographs of the same feet without apparent affiliation were presented again. The terms “normal,” “borderline normal,” and “abnormal” were assigned by the observers. Viewers considered MTP-2 alignment from 0° to −20° to be normal, and below −30° abnormal; MTP-3, 0° to −15° to be normal and below −30° abnormal; MTP-4, 0° to −10° normal and below −20° abnormal. Between 5° valgus and 15° varus was the range of MTP-5 recognized as normal. High intra-observer but low interobserver reliability with overall low correlation of clinical and radiographic aspects was observed. The assessment of the terms “normal” or “abnormal” are subject to a high degree of variation. Therefore, these terms should be used cautiously.

## 1. Introduction

The geometry of the entire foot and, in particular, the forefoot, is subject to a high degree of variation [1]. Genetic factors, depending on age, gender, and origin, have just as much influence as respective constitution, hormone status, body mass index, and traumas that may have taken place [1,2,3].

Even in ancient times, special attention was paid to the shape of the toe alignment. In addition to the Roman foot shape, in which the big toe and the second toe are of equal length, and the Egyptian foot shape, where the second toe is shorter than the big toe, the Greek foot shape has been awarded particular mention: the second toe is longer than the big toe. This foot shape is especially widespread in Europe, wherein almost every third person has a Greek foot shape. In ancient times, this was considered a special ideal of beauty, as the Greek goddesses are said to have possessed this foot shape [4,5].

To be distinguished from this are age-typical changes of the foot and toes. In addition to hallux valgus and claw and hammer toe deformities, the disturbed metatarsal parabola, with corresponding transfer metatarsalgia, is one of the typical foot complaints of the elderly [6,7,8]. The indication for surgical intervention for these changes is based on the level of distress and mostly on radiographic imaging in the weight-bearing dorsoplantar view.

The clinical description of the preoperative condition is based on terms such as “disturbed toe alignment or abnormal alignment.” The terms “normal” or “abnormal” are usually equated with “harmonious” and “inharmonious” [9]. The same applies to the evaluation of the surgical result where, for example, after a Weil osteotomy has been performed, the expressions “restoration of a harmonious parabola” or a “normal toe alignment” are regularly used [10,11]. Although experienced colleagues in foot surgery may develop a realistic impression from such condition descriptions, an objectifiable value of the respective metatarsophalangeal angles (MTPAs) 2–5, which justifies the use of “normal” or “abnormal,” is still missing [6]. We aimed to determine which MTPAs 2–5 are considered to be “normal” by orthopedic surgeons, especially those in foot surgery and radiology.

## 2. Materials and Methods

### 2.1. Population

Between 2022 and 2023, this multicenter study enrolled 30 patients (mean age 55 ± 17 years) with clinical images and corresponding radiographs, including 10 images each with presumed normal, borderline, or abnormal alignment. The mean first metatarsophalangeal angle (MTP-1A) of all patients was 19.6 ± 17.3°. The mean 1-to-2 and 1-to-2 intermetatarsal angle (M1-M2A) was 10.4 ± 5.5°, and the mean 1-to-5 intermetatarsal angle (M1-M5A) was 26.3 ± 6.7°. There was an equal distribution between the cases with presumed normal and abnormal toe position, which is why a separate presentation is omitted (*p* > 0.05).

All patients were seen in the foot surgery clinic of the study centers. Pre-existing conditions were not queried separately. All procedures were performed in accordance with the 1964 Helsinki Declaration and its later amendments. The ethics committee of the institutional review board approved this study.

### 2.2. Inclusion and Exclusion Criteria

The minimum age was 18 years; there was no maximum age. Inclusion in the present study was regardless of underlying complaints in the foot surgery clinic. Written informed consent was required prior to participation. Only clinical images of and radiographs of the weight-bearing foot were included in the study for evaluation. Any surgery on the affected toe or metatarsal being evaluated led to exclusion of the patient.

### 2.3. Observers

Five observers from four different hospitals performed a blind analysis in random order. The observers consisted of two residents in orthopedic surgery, two fellowship-trained foot surgeons, and one radiologist who specialized in musculoskeletal radiology.

### 2.4. Experimental Setup

**Figure 1:**
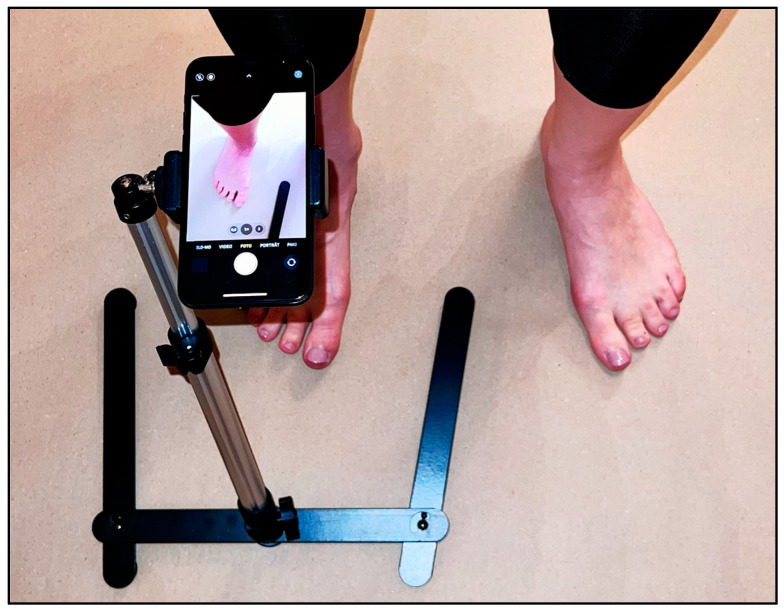
Experimental Setup.

**Figure 2:**
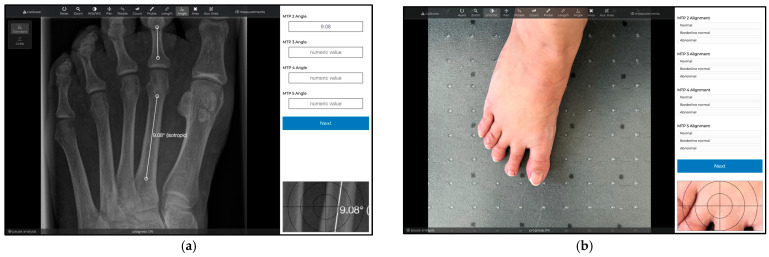
Web-based online analysis tool Tyche™. (**a**) Assessment of clinical images, (**b**) assessment of radiographic images.

The clinical images were collected based on standardized photographs, taken using an iPhone (Apple™, iOS 16.0.3), on the weight-bearing foot during the consultation hour, irrespective of the symptoms presented. For this purpose, a tripod was always positioned 30 cm centrally above the weight-bearing foot (Figure 1). Associated weight-bearing radiographs were also collected in a dorsoplantar (DP) view. After receiving approximately 80 images each, the authors pre-sorted the images into three groups: Presumptive Normal, Borderline Normal, and abnormal alignment. The images were then fed into a web-based online analysis tool (Tyche™) in anonymized and random order (Figure 2).

### 2.5. Assessment Methods

Five orthopedic surgeons analyzed 30 weight-bearing radiographs and corresponding photographs of the feet, twice. The observers first determined meta-tarso-phalangeal (MTP) angles of two, three, four, and five on the radiographs. They then individually scored MTP alignments of the second, third, fourth, and fifth toes on radiographs and corresponding photographs into the three categories. Angle measurements and alignment assessments on radiographs and photographs were carried out separately. The analysis was performed blinded, with images appearing in random order using recently introduced software, Tyche™ 1.0 (Figure 2). Observers were not provided with sample images for what should be considered “normal” or “abnormal” but were instead encouraged to give their personal ratings. The only evaluation criterion was personal experience in the respective field of surgery or radiology. Exact standard values for the MTP 2–5 angles have not yet been defined, therefore no objectifiable criteria for evaluation exist.

To facilitate a multicenter study including observers from different orthopedic hospitals, the recently introduced online tool, Tyche™, was utilized as follows [12,13]. Tyche™ functions as a survey tool that displays scientific images anonymized inside a web-browser along with standard tools for image analysis. On the same window, results can be stored in customizable input forms, such as text-fields or single-choice questions. Access to the images can be provided to the observers via temporarily valid URLs. Once the analysis is finished, results are merged and visible to the project manager.

### 2.6. Statistical Analysis

The number of cases required for this study is based on the following power assumptions: with an estimated 10 images per group (normal, borderline normal, abnormal), confidence intervals were estimated at 0.42 SDs, 0.85 SDs or 0.64 SDs, with a power of 80%.

As described by Popovic et al., we chose the following approach to estimate measurement accuracy: for every measurement on every toe, the standard deviation between the five observers was calculated [14]. Then, an average of these standard deviations was calculated and termed the mean of individual standard deviations. A low value, relative to the absolute result of a measurement, indicated high accuracy.

For categorial, nonmetric data, the inter- and intra-observer agreement was calculated using Fleiss’ kappa. Coefficients above 0.81 were considered as “almost perfect”; above 0.61, “substantial”; above 0.41, “moderate”; and above 0.21, “fair” (Table 1) [15]. For metric data, the inter- and intra-observer agreement was determined using intra-class correlation (ICC) coefficients with absolute agreement. Values <0.5 were interpreted as “poor reliability”; >0.5, moderate reliability; >0.75, good reliability; and >0.9, excellent reliability (Table 1) [16]. Statistical analysis was performed using SPSS 27.0 (IBM, Armonk, NY, USA) and Prism 9.4 (GraphPad Software, San Diego, CA, USA).

## 3. Results

**Figure 3:**
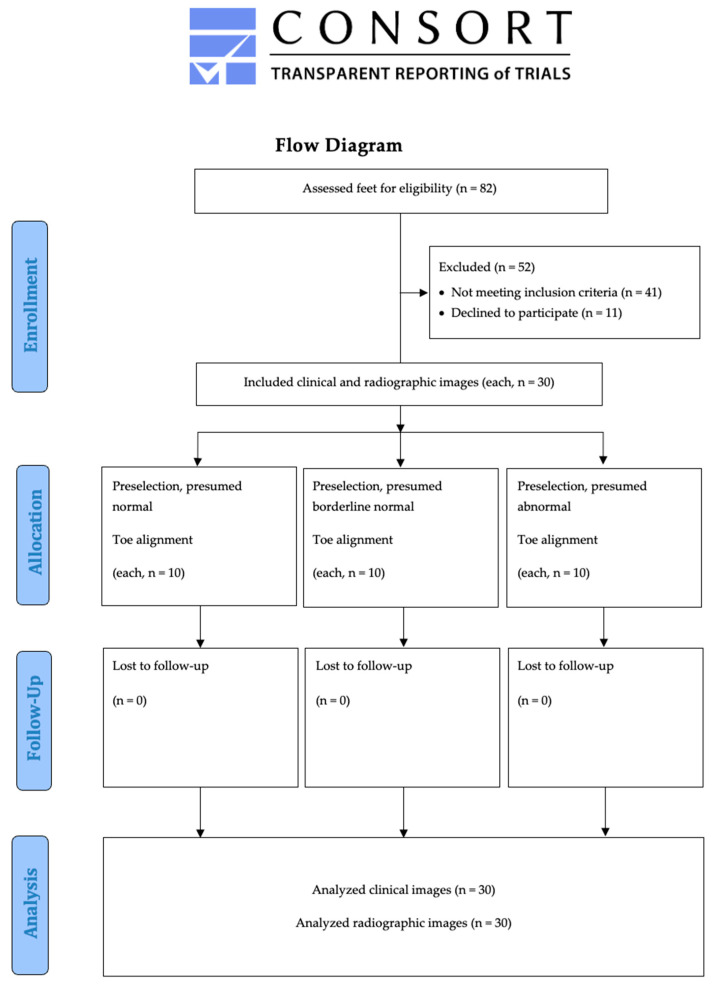
Study flow chart.

Thirty clinical and 30 radiographic logical images were analyzed with the following result (Figure 3): mean ± SD angles across all images were as follows: MTP-2, −12.4 ± 17.7°; MTP-3, −16.8 ± 15.3°; MTP-4, −11.9 ± 13.7°; and MTP-5, 3.2 ± 16.7°. The angle measurement inter- and intra-observer reliability were calculated using ICCs and ranged from 0.95 to 0.99, indicating excellent reliability. The means of individual standard deviations between the observers were used to estimate the accuracy of a single measurement, and ranged from 2.3° for MTP-4 to 2.8° for MTP-5 (Table 2).

Five observers measured MTP angles 2, 3, 4, and 5. Positive values indicate varus alignment, and negative values indicate valgus alignment. Results are shown with overall SDs. Inter- and intra-observer reliability were determined using ICC coefficients and were all above 0.95. Standard deviations were determined for every measurement on every image between all five observers. The mean of these SDs across all images ranged between 2.3° to 2.8°. Low values indicate high accuracy of an individual measurement.

Fleiss’ Kappas were calculated to determine inter- and intra-observer reliability regarding the alignment assessments for radiographs and photographs. On radiographs, interobserver reliability was poor for MTP-4 (0.48) and MTP-5 (0.27) alignment and moderate for MTP-2 (0.65) and MTP-3 (0.52) alignment. In comparison, intra-observer reliability was consistently higher, showing moderate reliability, for MTP-3 (0.73) and MTP-4 (0.66) alignment and even good reliability for MTP-2 (0.76) and MTP-5 (0.8) alignment (Table 3).

Fleiss’ kappa was used to determine inter- and intra-observer reliability of the grading scores. MTP-5 interobserver alignment had the lowest reliability. Intra-observer reliability was higher across all angles than interobserver reliability. On photographs, interobserver reliability was consistently poor for all angles (0.08–0.46), and intra-observer reliability was higher for all angles (0.56–0.71), indicating at least moderate agreement (Table 4).

The same assessment, as shown in Table 3, was performed by applying the identical three-graded score on photographs facing the dorsum of the barefoot. Fleiss’ kappa was again used to determine inter- and intra-observer reliability. MTP-4 interobserver alignment had the lowest reliability. Intra-observer reliability was higher across all angles than interobserver reliability.

Alignment assessments from radiographs were compared with those from corresponding photographs from the same feet for all observers using Fleiss’ kappa values (Table 5). Except for Observer V for MTP-3 alignment, which was moderate (0.58), all other observers showed only poor reliability.

Each observer applied a three-grade score (see Table 3 and Table 4) to assess MTP alignment on radiographs and photographs of the same foot. Fleiss’ kappa was used to determine the correlation between the assessments performed on radiographs and photographs for each observer. The last row shows the mean coefficients from all five observers. Values ranged from 0.16 to 0.37.

**Figure 4:**
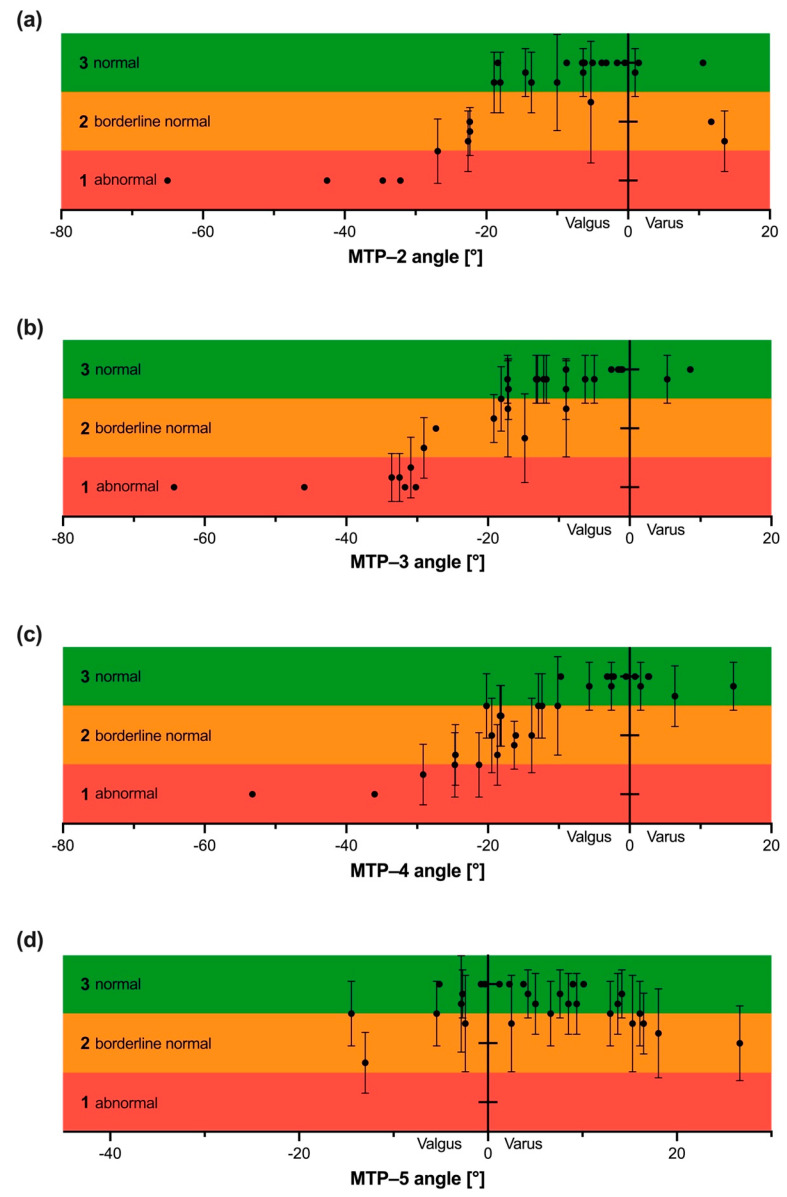
Six experts measured MTP–angles two (**a**), three (**b**), four (**c**) and five (**d**) on thirty dorsoplantar (DP) radiographs. Results were plotted on the X–axis. In addition, they graded the MTP–alignment, based on their personal opinion, into three grades: “normal”, “borderline normal” and “abnormal”. An ordinal scale was created giving “normal” the number 1, “borderline normal” the number 2 and “abnormal” the number 3. A mean value with SD was calculated between the six experts for every MTP joint and plotted on the Y–axis. This can be considered the “mean assessment” between the six experts. SD, standard deviation.

Finally, mean MTP angles with alignment assessments from all observers, and a mean assessment with SDs between the observers was calculated. MTP-2, -3, and -4 show a similar sigmoidal pattern with an abnormal alignment toward high valgus deviations and a normal alignment toward 0° deviation. While the same feet have more data points on valgus alignment for MTP-2, -3, and -4, MTP-5 has more data points for varus alignment (Figure 4).

## 4. Discussion

Based on the data obtained, the clinical impression of the authors is confirmed that, despite comparably excellent determination (ICC > 0.9) of the respective MTP 2–5 angles by the different observers, the assessment of whether the foot has normal or abnormal lesser toe alignment differs widely. This is applied equally to the assessment of the clinical images and the associated radiographs. However, the multi-time assignments of the respective observers were little consistent, with a Fleiss’ kappa of 0.66 to 0.8, defined as “substantial” [15].

Again, all the results from our observers equally lack a comprehensible correlation between clinical photographs judged to be normal or abnormal.

Interobserver agreement in assessing toe alignment from photographs was worse than when assessed from radiographs. However, the consistency of the individual measurements was better. The fact that the examiners were very consistent in their assessment, despite the time interval and randomized presentation of the images, indicates a sound assessment ability. This was observed regardless of the specialty.

Figure 4 shows “mean assessments” between six observers for different MTP-angles. At certain ranges, there is a low SD and a high agreement between the observers can be concluded. Based on these ranges, with high agreement on the alignment, the following conclusions were made, yet they remain objective: MTP-2 alignment is normal from 0° to −20° and abnormal below −30°; MTP-3 alignment is normal from 0° to −15° and abnormal below −30°; MTP-4 alignment is normal from 0° to −10° and abnormal below −20°. The “borderline range” varies between these values, where the largest uncertainty in inter- and intra-observer reliability was found. The range of MTP-5 recognized as normal was between 5° valgus and 15° varus.

The available data confirm the underlying assumption of the study that the same photos and radiographic images were assessed differently, even by experienced colleagues, regardless of whether an orthopedist or radiologist made the assessment. It can be summarized that, regardless of the terminology “normal” and “abnormal” in the assessment of toe alignment, a reliable conclusion from the clinical aspect or photo to the radiograph, and vice versa, is not possible.

Although numerous radiological normal values are available for the foot, especially for the first ray, the overall definition of a “normal foot” remains no less difficult than the definition of normal “toe alignment” [17,18,19]. Even with the help of qualitative and semi-quantitative visual appraisal, anthropometric measurements, and footprint analysis, only approximate standard values for the clinical and radiological aspects of the foot can be found [20].

In addition, even putative pathological configurations do not necessarily correlate with reduced performance or even pain [21]. This is shown by studies of various foot deformities in sports, which, for example, attribute to a pronounced flatfoot deformity in children and adults, approximately the same performance as clinically and radiologically supposedly normatively configured feet [22,23].

This is similar for the presumed normal parabola of the metatarsalia, whereby the protrusion of the second metatarsalia is of comparable importance to the 1-to-2 and 1-to-5 intermetatarsal angle (M1-M2A and M1-M5A) in the radiographic evaluation of forefoot alignment [24]. These values are important to understand the biomechanics of the forefoot and determine the normal shape and function of the foot during orthopedic and surgical treatment [6]. These findings can indeed demonstrate gender-specific differences in forefoot alignment. However, no conclusions can be drawn about the performance of the foot, nor about when a configuration should be considered abnormal or even pathological [6].

To minimize the influence of an underlying forefoot malalignment on the lesser toe alignment, care was taken to ensure that at least the M1-M2A and M1-M5A was close to the normal range and the hallux valgus (HV) angle was equally distributed between the groups [25,26,27]. With a mean talo-1-MT1 angle, the so-called hallux valgus angle, of 19.6°, the measurements deviate only slightly from the normal range and can be regarded as age-typical changes at an average age of 55 years.

In orthopedics and trauma surgery, pathological and normal findings are usually defined by objective criteria. These are currently only available to a limited extent when considering the MTP 2–5 angles. The results of the present study with the first definition of normal and abnormal MTP 2–5 angles should be compared with the respective complaints of patients in subsequent studies.

There are some limitations to mention. All assessments of the clinical and radiographic images are subject to the bias of preselection. The presentation, for example, of configurations that could be evaluated exclusively as certainly abnormal could have led to a deviating assessment. Another limitation could be found in the toe malalignment itself. Despite standardized imaging and radiographic techniques, hammer and claw toe deformities may lead to distortion of the true aspects of the foot.

## 5. Conclusions

The assessment of the terms “normal” or “abnormal” with regard to toe alignment is subject to a high degree of variation, even among specialized foot surgeons and radiologists. In particular, the assessment of clinical images was significantly different between observers. Therefore, the terms normal or abnormal should be used cautiously when describing the clinical aspect, especially when evaluating a surgical result based on the radiographs in the DP view. Rather, the joint function and the clinical outcome should be considered.

## Figures and Tables

**Table 1:** Interpretations for Fleiss’ kappa and Intra-Class Correlation coefficients (ICC).

Fleiss’ Kappa	Interpretation ^a^	ICC	Interpretation ^b^
>0.81	Almost perfect	>0.9	Excellent
>0.61	Substantial	>0.75	Good
>0.41	Moderate	>0.5	Moderate
>0.21	Fair	<0.5	Poor

^a^ Landis & Koch [15], ^b^ Koo & Li [16].

**Table 2:** MTP-2, -3, -4, and -5 angles with reliability analysis.

	MTP-2	MTP-3	MTP-4	MTP-5
Mean ± SD	−12.4 ± 17.7°	−16.8 ± 15.3°	−11.9 ± 13.7°	+3.2 ± 16.7°
Interobserver ICC	0.98 *	0.97 *	0.96 *	0.95 *
CI	0.96–0.99	0.94–0.98	0.93–0.98	0.92–0.98
Intra-observer ICC	0.99 *	0.99 *	0.99 *	0.99 *
CI	0.99–0.99	0.98–0.99	0.98–0.99	0.99–0.99
Mean of individual SDs	2.4°	2.5°	2.3°	2.8°

MTP, metatarsophalangeal angle; SD, standard deviation; ICC, intra-class correlation coefficient; CI, confidence interval, * *p*-value < 0.001.

**Table 3:** Reliability of alignment assessment on radiographs.

	MTP-2	MTP-3	MTP-4	MTP-5
Interobserver Kappa	0.65 *	0.52 *	0.48 *	0.27 *
Confidence interval	0.60–0.70	0.48–0.57	0.44–0.53	0.22–0.23
Intra-observer Kappa	0.76 *	0.73 *	0.66 *	0.8 *
Confidence interval	0.66–0.87	0.62–0.83	0.55–0.77	0.67–0.92

MTP, metatarsophalangeal angle, * *p*-value < 0.001

**Table 4:** Reliability of alignment assessment on photographs.

	MTP-2	MTP-3	MTP-4	MTP-5
Interobserver Kappa	0.46 *	0.38 *	0.26 *	0.08 *
CI	0.40–0.51	0.32–0.43	0.20–0.32	0.01–0.16
Intra-observer Kappa	0.71 *	0.64 *	0.68 *	0.56 *
CI	0.60–0.82	0.53–0.76	0.57–0.80	0.41–0.71

MTP, metatarsophalangeal angle; CI, confidence interval, * *p*-value < 0.001

**Table 5:** Correlation of assessments between radiographs and photographs.

		MTP-2	MTP-3	MTP-4	MTP-5
Observer I	Kappa	0.26 *	0.37 *	0.15	0.20
	CI	0.08 to 0.44	0.19–0.55	−0.04 to 0.33	0.00 to 0.39
Observer II	Kappa	0.14	0.24 *	0.05	0.12
	CI	−0.05 to 0.32	0.06 to 0.42	−0.13 to 0.23	−0.1 to 0.35
Observer III	Kappa	0.44 *	0.46 *	0.03	0.09
	CI	0.26 to 0.63	0.27 to 0.64	−0.16 to 0.23	−0.15 to 0.32
Observer IV	Kappa	0.34 *	0.40 *	0.2 *	0.27 *
	CI	0.16 to 0.52	0.21 to 0.85	0.01 to 0.39	0.04 to 0.49
Observer V	Kappa	0.33 *	0.58 *	0.38 *	0.34 *
	CI	0.15 to 0.52	0.40 to 0.77	0.20 to 0.57	0.10 to 0.58
Mean	Kappa	0.30	0.37	0.16	0.20

MTP, metatarsophalangeal angle; CI, confidence interval, * *p*-value < 0.001

## Data Availability

All data intended for publication are included in the manuscript.
